# Advancing typhoid conjugate vaccine implementation in Asia: Regional policy priorities

**DOI:** 10.1016/j.vaccine.2025.127848

**Published:** 2025-10-14

**Authors:** Alice S. Carter, Duncan Steele, Jacob John, Senjuti Saha, Matthew B. Laurens, Anna A. Minta, Litiana Volavola, Ismoedijanto Moedjito, Kongxay Phounphenghack, Diana Mahat, Soe Lwin Nyein, Abhiyan Gautam, Maria Fe Viviane S. Sespeñe, Teuila Pati, Nguyen Thi Thu Huong, Nivedita Gupta, Leyanna Susan George, Madhumathi Jayaprakasam, Anuradha Gupta, Denise O. Garrett

**Affiliations:** a Applied Epidemiology, Global Immunization, Sabin Vaccine Institute, Washington, DC, USA; b Enterics, Diagnostics, Genomics & Epidemiology, Gates Foundation, Seattle, USA; c Christian Medical College, Vellore, India; d Children’s Health Research Foundation, Dhaka, Bangladesh; e Center for Vaccine Development and Global Health, University of Maryland School of Medicine, Baltimore, MD, USA; f Global Immunization Division. US Centers for Disease Control and Prevention, Atlanta, GA, USA; g Ministry of Health and Medical Services, Suva, Fiji; h National Immunization Technical Advisory Group, Jakarta, Indonesia; i National Immunization Program, Vientiane, the Lao People’s Democratic Republic; j Ministry of Health, Kuala Lumpur, Malaysia; k National Immunization Technical Advisory Group, Naypyidaw, Myanmar; l National Immunization Program, Department of Health Services, Ministry of Health and Population, Kathmandu, Nepal; m Department of Health, Manila, Philippines; n Ministry of Health, Apia, Samoa; o National Institute of Hygiene & Epidemiology, Hanoi, Viet Nam; p Indian Council of Medical Research, New Delhi, India

**Keywords:** *Salmonella typhi*, typhoid, typhoid conjugate vaccine, vaccine introduction

## Abstract

Typhoid fever, a type of enteric fever transmitted through consumption of food or water contaminated with *Salmonella enterica* subspecies Typhi, continues to cause illness in Asia. Since the World Health Organization recommended use of typhoid conjugate vaccines in 2018, several countries have introduced or begun the decision-making process to use the vaccine in campaigns or by introduction into the routine immunization schedule. This paper describes the disease burden and vaccine implementation policy setting in the region, based on presentations and discussions at the second Asia Regional Meeting on Typhoid & TCV. Moving forward, typhoid control efforts must prioritize 1) working across health and finance ministries to finance vaccination programs, 2) strengthening surveillance to better understand disease burden, drug resistance trends, and vaccine impact, 3) developing improved diagnostic tests, 4) stewarding antimicrobial use to slow the spread of drug resistance, and 5) evaluating the durability of TCV protection.

## 1. Introduction

**Fig. 1.**
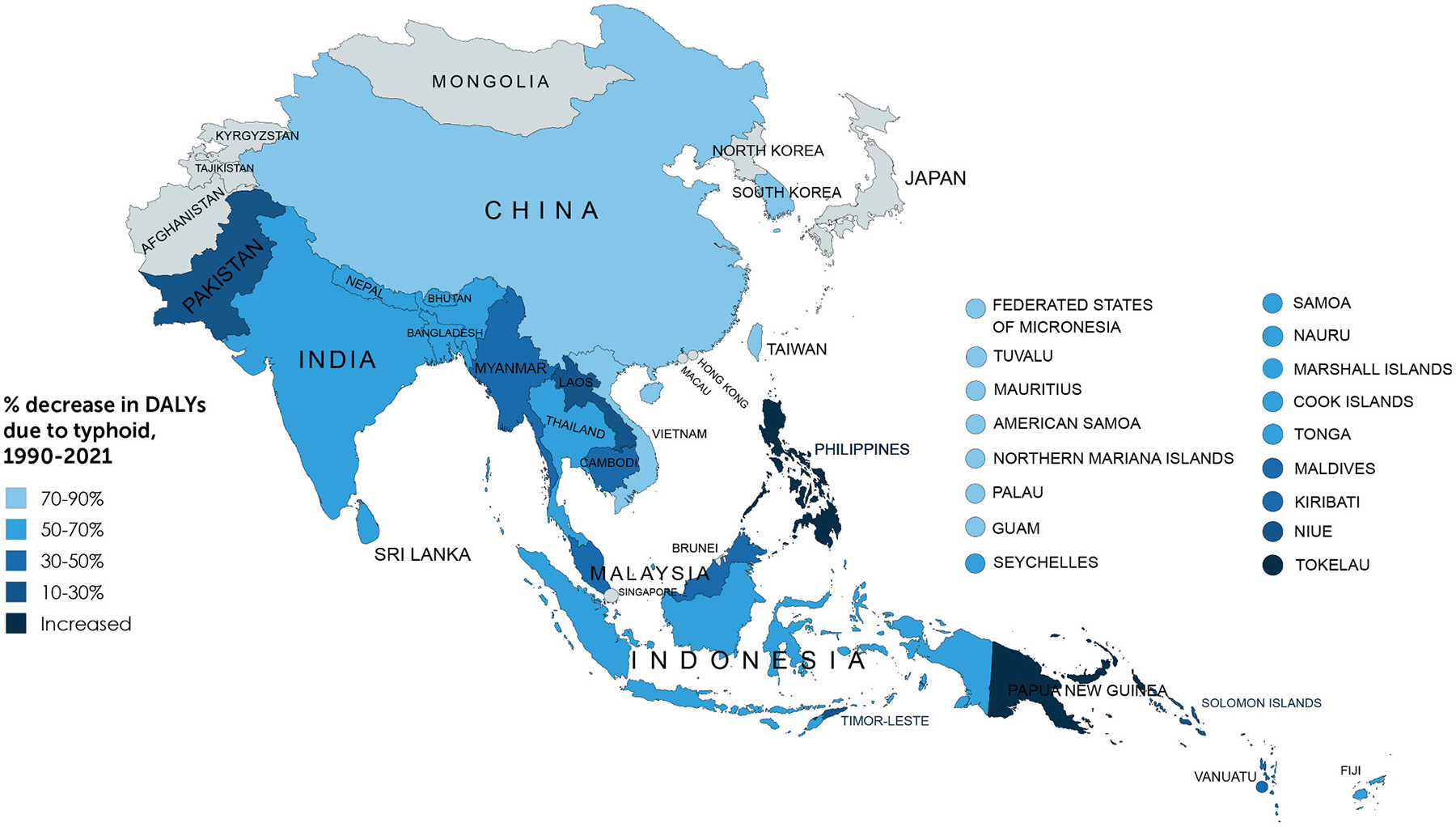
Reduction in typhoid cases in Asia and Oceania, 1990–2021. Map showing percent decrease in DALYs attributable to typhoid in the South Asia and Oceania regions over the 1990–2021 period. Data from the Global Burden of Disease Study. [43]

Typhoid, a bacterial illness transmitted through contaminated food or water, remains a major public health threat in Asia, where 70 % of the global cases occur (Fig. 1). [1] Each year, typhoid causes an estimated 5.1 million cases and more than 72,000 deaths in the region, with children under 15 years of age at highest risk. [2]

Since 2018, the World Health Organization (WHO) has prequalified and recommended use of typhoid conjugate vaccines (TCV) in countries with the highest burden of typhoid disease or a high burden of drug resistant *Salmonella Typhi*. [3] Despite TCV availability and demonstrated efficacy, only a handful of countries in Asia and Oceania – Nepal, Pakistan and Samoa – have introduced TCV. Several other countries are planning or considering introduction, including Bangladesh, India and Indonesia (Table 1).

To coordinate action and advance regional dialogue, the Sabin Vaccine Institute and the Indian Council of Medical Research (ICMR) convened the 2024 Asia Regional Meeting on Typhoid & TCV from August 27–28, 2024, in New Delhi, India. The meeting gathered approximately 75 key stakeholders, including immunization managers, Ministry of Health representatives, national immunization technical advisory group (NITAG) members, and researchers from 13 countries – Bhutan, Fiji, India, Indonesia, Lao PDR, Malaysia, Myanmar, Nepal, Philippines, Samoa, Sri Lanka, and Vietnam – alongside representatives from WHO headquarters and Southeast Asia Regional Office (SEARO), the US Centers for Disease Control and Prevention, and the Typhoid Vaccine Acceleration Consortium (TyVAC).

The two-day meeting included oral presentations on the regional typhoid burden and global control efforts (Day 1), followed by working group discussions to share lessons learned (Day 2). This report summarizes the key discussions to inform global policy decisions and regional vaccine introduction.

### 1.1. Typhoid burden in Asia

Jacob John highlighted the persistently high typhoid burden in South Asia, especially in urban areas. [4] Surveillance projects such as Surveillance for Typhoid in Asia Project (SEAP), TyVAC, and Surveillance for Enteric Fever in India (SEFI) consortium indicate high incidence rates in Bangladesh, India, Nepal, and Pakistan. [5–8] Young children are at the highest risk, but the risk of illness persists into adulthood. Challenges include unreliable point-of-care diagnostics, under-detection of cases due to limited blood culture availability and sensitivity, and rising resistance to frontline antimicrobials such as azithromycin, ceftriaxone and ciprofloxacin [9].

Venkata Raghav Mohan (Christian Medical College, Vellore, India) presented wastewater monitoring and seroincidence estimation as emerging tools to enhance national typhoid surveillance. Both techniques address the limitations of blood culture – a low-sensitivity diagnostic that is difficult to sustain in low-resources settings. For example, environmental surveillance was recently used in urban Vellore to estimate subclinical disease transmission within communities, finding an overall positivity rate of 11.4 %. [10] A novel serological assay that can be used to measure antibody kinetics over time found a seroincidence rate in Vellore of 10.4 cases per 100 person-years. [11]

Senjuti Saha warned of the escalating antimicrobial resistance (AMR) crisis, with drug-resistant *Salmonella* strains spreading rapidly. [12] Currently, 80 % of enteric fever cases are treated with oral antimicrobials – typically azithromycin – in an outpatient setting; these public health clinics could not manage an influx of typhoid cases requiring injectable treatment. Worryingly, azithromycin resistance is caused by a single point mutation and can occur in multiple serovars, making it a serious public health threat.

### 1.2. Vaccines

Matthew Laurens provided updates on TCV introduction and impact. Four TCVs have been prequalified by WHO. [13] Efficacy studies conducted in Africa and Asia demonstrated 79–85 % efficacy over at least one to four years of follow-up and demonstrated that TCV can be safely co-administered with other routine childhood vaccines. [14–18] Modeling data presented at the meeting demonstrated that under all circumstances, introduction of TCV will be cost-effective and cost saving in urban areas of India. [19]

**Fig. 2.**
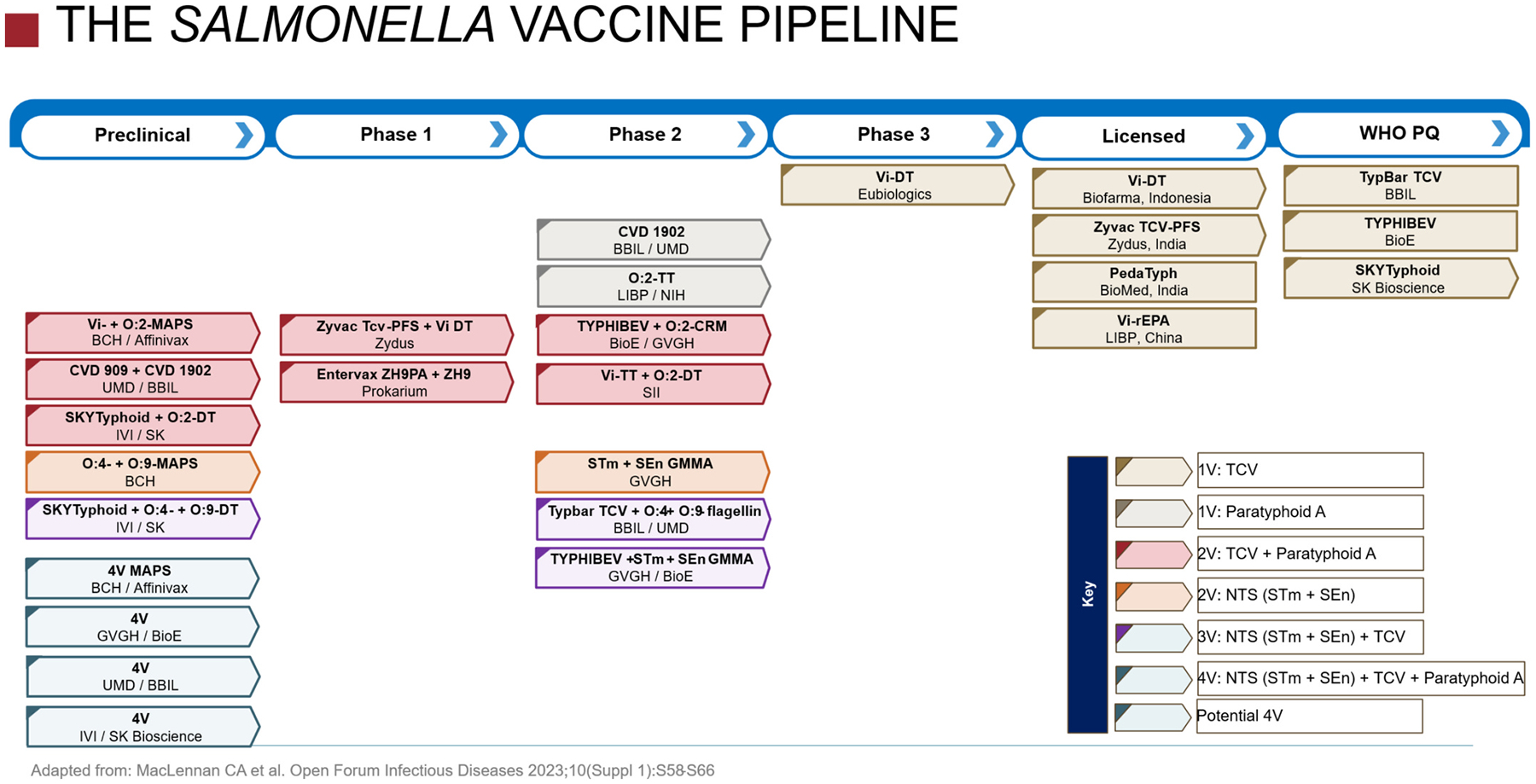
The *Salmonella* vaccine pipeline. Pipeline of candidate vaccines against typhoid, paratyphoid, and nontyphoidal *Salmonella*. [20]

Duncan Steele outlined additional vaccines in the development pipeline (Fig. 2). Currently, nine candidates – including bi-valent and tri-valent vaccines against typhoid, paratyphoid, and multiple invasive non-typhoidal *Salmonella* (iNTS) strains – are in clinical trials. An additional nine vaccine candidates, including quadrivalent vaccines, are in the pre-clinical development stage. [20] Given global incidence rates of 91 cases of typhoid per 100,000 person-years, 27 cases of paratyphoid per 100,000 person-years, and 6 iNTS cases per 100,000 person-years, combination vaccines could offer broadened protection against enteropathogens that can be difficult to distinguish clinically. [2] The WHO recently published Preferred Product Characteristics for new *Salmonella* vaccines, recommending that new candidates demonstrate non-inferiority of the typhoid Vi component against currently licensed TCVs, safety and immunogenicity of the paratyphoid O2 conjugate component, and evidence of clinical protection via controlled human infection models with immune-bridging from adults to children. [21]

### 1.3. Introducing TCV

Eileen Quinn (PATH, USA) stressed the need for early engagement in TCV introduction planning. The full process – from NITAG decision-making to securing Gavi funding and finalizing rollout logistics – can take two years or more. Countries are encouraged to initiate discussions early, so that vaccine subsidy support can be provided during their Gavi eligibility window. Postponing vaccine introduction increases avoidable morbidity and ongoing transmission of increasingly drug-resistant typhoid. Even when treatable, typhoid imposes catastrophic financial burdens on families. Moreover, as climate change-related events worsen, the risk for typhoid outbreaks and spread increases.

Anna Minta presented WHO resources for typhoid surveillance and TCV introduction. WHO envisions comprehensive, high-quality, sustainable surveillance systems in all communities. [22] The 2018 surveillance standards for typhoid and other invasive salmonellosis recommend laboratory-based, facility-based surveillance as a minimum in typhoid endemic settings, which is ideally integrated into the existing health reporting system. [23,24] To further enhance surveillance, WHO is developing a laboratory manual for typhoid and paratyphoid diagnosis and a Target Product Profile (TPP) for acute typhoid fever surveillance using rapid diagnostic tests or enzyme immunoassays. [25] However, lack of routine typhoid surveillance should not be a barrier to TCV introduction. As recommended by the WHO Strategic Advisory Group of Experts on Immunization (SAGE), any country with evidence of typhoid disease burden or evidence of high burden of antimicrobial resistant *S. typhi* is encouraged to consider TCV introduction.

### 1.4. Country updates

(see Table 2).

#### 1.4.1. Bangladesh

Bangladesh faces a high burden of typhoid, with an estimated incidence rate of 913 cases per 100,000 person-years in Dhaka. [5] Limited surveillance data comes from research programs rather than national surveillance systems. For example, Bangladesh has had ongoing research-funded typhoid surveillance that identified an outbreak of ceftriaxone-resistant typhoid in May 2024 that continues to spread. [9] The NITAG recommended TCV introduction in 2023, with further urgency added by the XDR outbreak, and introduction is planned for 2025. To secure the NITAG recommendation for introduction and government-wide support, cooperation from ministerial counterparts and agencies, especially the education, water, and finance ministries, was critical.

#### 1.4.2. Bhutan

Tashi Dawa, EPI Program Manager, presented on behalf of Bhutan. Bhutan’s typhoid burden estimates come from their sentinel surveillance of diarrheal diseases program. Recent data shows about nine diagnosed cases of typhoid per year. The stool culture positivity rate has decreased from a high of 5.7 % in 2021 to an estimated 1.1 % in 2024. Bhutan has achieved 99.7 % coverage of access to safe drinking water and achieved open defecation free status in 2022. Due to low case counts, typhoid vaccine introduction remains low priority. However, neither diarrhea surveillance nor stool culture are recommended for typhoid surveillance, and the country is encouraged to consider blood culture or serological assays as a more reliable indicator. Across the board, limited data on emerging and re-emerging diseases complicates vaccine introduction decisions.

#### 1.4.3. Fiji

Tiko Saumalua and Litiana Volavola presented updates from the Republic of Fiji. The surveillance system, based at three tertiary care hospitals, has seen an average of 270 cases per year in the last five years, with an estimated 2024 incidence rate of 31 cases per 100,000 person-years.

The Fijian government considers typhoid control a high priority and implemented vaccine as a bridge to WASH. Fiji’s TCV campaign required significant outreach and communication resources, in part to overcome vaccine hesitancy following the COVID-19 outbreak. The introduction campaign prioritized minimizing vaccine wastage and targeted vaccinating 300,000 people. The program invested heavily in community-based communications and outreach to increase coverage from the 50 % achieved in the initial campaign.

Challenges faced by Fiji’s typhoid program include outbreaks presumably resulting from asymptomatic shedders. Additionally, TCV was introduced at a new 9-month vaccination visit, which presented a burden for families; the EPI may shift the TCV to be offered at the existing 12-month visit. Fijian health policymakers request training and programmatic resources, such as guidelines for vaccine use from the WHO, to support the continued TCV campaign. For example, guidelines would provide necessary support on cold chain implementation, laboratory diagnostics supply chain, and continued training for vaccinators to increase vaccine uptake.

#### 1.4.4. India

Typhoid is a high burden disease in many Indian cities, and blood culture surveillance indicates that typhoid is the leading cause of community-acquired bacteremia. One in six people in Vellore will develop typhoid, as will one in ten in Kolkata and one in twelve in Delhi. [8] Surveillance has demonstrated emerging AMR to quinolones, macrolides, and third-generation cephalosporins. Over-the-counter antibiotic use is widespread and 40–60 % of patients presenting at the clinic already have taken antibiotics, so blood culture positivity is likely an under-estimate of the true case burden.

Effectiveness of TCV was evaluated in Navi Mumbai in a mass vaccination program for children 9 months to 14years from September 2018 to July 2020. The vaccine effectiveness of 83.7 % (95 % CI: 45.0–95.3) was similar to prior randomized controlled trials and provides reassurance to policymakers that TCV effectiveness is robust in a large-scale implementation program managed by the routine immunization system. [26] Cost-effectiveness estimates indicate that introduction of TCV is a cost-saving strategy in urban India. [19]

India’s NITAG decided in 2022 to introduce TCV and secured a commitment of Gavi introduction support. They also recommended establishing typhoid surveillance to determine disease burden, plan control strategies, target the vaccination strategy, monitor the impact of vaccine campaigns on disease epidemiology, monitor AMR, inform treatment and control, detect outbreaks, determine the need for boosters, coordinate disease elimination, and evaluate a potential bivalent vaccine switch. External research has highlighted additional evidence priorities to support TCV decision-making in India, including public perception of typhoid and TCV, vaccination budget impact and fiscal space, vaccine availability, and socioeconomic impact. [27]

#### 1.4.5. Indonesia

Ismoedijanto Moedjito presented a report on the typhoid situation in Indonesia. Indonesia’s typhoid burden has not decreased in recent years. However, in the past few years, Indonesia has implemented public health interventions such as increasing access to safe water and health education programs that may impact disease transmission.

TCV is considered a medium-priority vaccine in Indonesia. Key challenges to TCV introduction include budgetary concerns and chronically low immunization coverage in the routine immunization program. The public health system is also focused on large areas at high risk of polio outbreaks as well as outbreaks of diphtheria and measles. Indonesian policymakers face competing health priorities but are interested in building out their vaccine security through local manufacturing.

The representatives from Indonesia discussed the desire for local disease burden data, cost-of-illness data, and cost-effectiveness data to inform introduction decision-making and measure vaccine program impact. With adequate support, Indonesia could launch a TCV demonstration project in a highly endemic area to establish evidence of success.

#### 1.4.6. Lao PDR

Kongxay Phounphenghack presented country updates on behalf of Lao PDR, where typhoid is endemic in rural areas with poor sanitation and limited access to clean drinking water, and where treatment is complicated by emerging AMR.

Public health interventions like education programs and sanitation improvements during COVID-19 may have contributed to a reduction in the typhoid burden. In 2000, there were 443 typhoid cases per 100,000 person-years of observation. The burden estimate dropped to 13 cases per 100,000 person-years in 2017, and the estimates will again be updated in 2024. Inadequate laboratory surveillance complicates accuracy of the disease burden assessment, but public health leaders hope COVID-19 investments strengthened laboratory capacity.

Lao PDR is considering introduction of rotavirus, diphtheria-tetanus booster and typhoid vaccines. TCV introduction is considered low-priority for the general population and moderate-priority for higher risk groups and in high-burden geographic areas. Higher prioritization is challenged by a lack of budget and a lack of nationwide data.

#### 1.4.7. Malaysia

Diana Mahat presented country updates from Malaysia. Malaysia, where typhoid is a mandatory notifiable disease, has recorded reductions in cases for the past ten years. In 2015, Malaysia had an incidence rate of 1.39 cases per 100,000 population (423 confirmed cases), dropping to a 2024 incidence rate of 0.42 cases per 100,000 population (143 confirmed cases).

Several public health interventions impacting the typhoid burden have been implemented. Malaysia has 94.9 % coverage of safe water and 99.7 % coverage of sanitation services. The government conducts hand washing and hygiene education and awareness campaigns, provides hand washing facilities with soap in public toilets and eateries with 99.9 % coverage, and enforces regulatory requirements for typhoid vaccination for food handlers.

Live attenuated and polysaccharide typhoid vaccines are available in the private sector for special populations including food handlers, lab workers, and sewage/sanitation workers. [28] In 2023, approximately 839,000 typhoid vaccines were administered. In 2024, 572,365 typhoid vaccines were administered. To strengthen its typhoid vaccine program, Malaysia requests WHO guidance for vaccination of low-incidence populations.

#### 1.4.8. Myanmar

Soe Lwin Nyein presented country updates from Myanmar, where the typhoid burden has not significantly declined in recent years. Previously, clinicians relied on empirical diagnosis, such as the unreliable Widal tests, or insensitive blood culture, resulting in misdiagnosis and under diagnosis of many children. Mathematical modeling suggests that in 2021 there were 83,309 cases of typhoid in Myanmar, indicating 148 cases per 100,000 population and 56 % of cases occurred in children less than 15 years of age. The same research indicates 1175 deaths from typhoid in 2021, with 65 % of deaths in children less than 15 years of age. [29] Another study found an annual incidence of 391 cases per 100,000 adolescents and adults in the Yangon region. [30] Prevention and control of typhoid outbreaks are a priority due to increasing risks of cyclones and floods, as well as earthquake disasters, WASH challenges, and lifestyle changes.

TCV is considered a high-priority and is included in the 2025–2029 national immunization strategy, with introduction planned in 2027. Myanmar has successfully improved its national surveillance system and laboratory capacity, and implementation of TCV introduction will depend on epidemiological data, financial support from Gavi, and continued political will.

#### 1.4.9. Nepal

Abhiyan Gautam presented country updates on typhoid and TCV in Nepal. Burden data are predominantly from urban and peri-urban Kathmandu, and cases are suspected to be under-reported from other areas. Research projects have found typhoid incidence rates ranging from above 400 cases per 100,000 person years to 1014 cases per 100,000 person years. [31] Different areas have highly variable incidence rates, with a ten-fold difference observed between communities. [5] Among all *Salmonella* isolates, 80 % are *S. typhi*. Environmental surveillance in Lalitpur found *S. typhi* in 77 % of all water samples collected.

Nepal vaccinated more that 7.7 million children in a community- and school-based typhoid campaign in 2022. A post-campaign coverage survey showed a national coverage rate of 84.1 %. After the campaign, TCV was introduced into the routine immunization program, where 2023 coverage was 98 %.

Gautam noted the need to elevate clean water and behavioral elements of typhoid control. Nepal introduced a collaborative program of “Hygiene Promotion Through Routine Immunization” in 2020 aiming to promote positive hygiene behaviors to decrease enteric diseases. He also highlighted that it may be challenging to measure the impact of TCV introduction in Nepal since the vaccine was introduced when cases were low during COVID. Notably, Nepal introduced TCV to be offered at 15 months, and high coverage is helping increase measles vaccine coverage at the same timepoint.

#### 1.4.10. Philippines

Maria Fe Viviane S. Sespeñe delivered an update on typhoid and TCV in the Philippines, where cases of typhoid appear to be climbing. In 2022, there were an estimated 15,873 cases and 63 deaths from typhoid, in 2023 there were an estimated 23,149 cases and 85 deaths, and in 2024 there were 29,249 cases and 88 deaths. These may be underestimates, since the widespread use of antibiotics results in low blood culture positivity and the health system misses cases presenting at first-level care centers. The Philippines uses Typhidot (Reszon Diagnostics) and Widal test for laboratory confirmation of typhoid because blood culture facilities are not widely available.

Challenges to prioritizing TCV introduction in the Philippines include cost and the low reliability of case estimates. In lieu of introducing TCV, the Philippines is currently relying on prevention of typhoid through sanitation and hygiene measures. For example, new mothers are offered educational programs on proper food handling in mothers’ classes. Food establishments and ambulant vendors are regulated with food handling training and health card permits.

The Philippines is developing a national immunization strategy and has focused efforts on the establishment of a NITAG. Immunization has been identified by the Secretary of Health as a priority, with a goal to increase the percentage of fully immunized children from 72 % to 95 %.

#### 1.4.11. Samoa

Teuila Pati delivered country updates on behalf of Samoa. TCV was introduced as part of a comprehensive typhoid elimination plan, including enhanced surveillance and public awareness campaigns. Vaccine was offered to all Samoans aged 1 to 45 years through a mass campaign, followed by introduction of the vaccine to the routine immunization program at 12 months of age. Concurrently, the country implemented laboratory testing for food handlers and sanitation improvements and drinking water chlorination campaigns in schools. Personal experiences drove political will to introduce TCV, as well as strong political pressure as typhoid cases were perceived to impact tourism and other business. Over five years, the country case counts fell from 100+ to fewer than 20 laboratory-confirmed cases annually.

Since TCV introduction, efforts have been focused on strengthening the overall immunization program and coverage levels. The country has faced technological challenges in assessing vaccine coverage due to a transition to an electronic health database system and programmatic challenges in reaching young adults. Nevertheless, through a rapid reduction of typhoid cases following vaccine introduction, TCV has been exemplary of the benefits of vaccination.

#### 1.4.12. Sri Lanka

Thilanga Ruwanpathirana, an epidemiologist with the Sri Lanka Ministry of Health, presented typhoid and TCV updates from Sri Lanka. Sri Lanka has typhoid burden data from laboratory-confirmed cases and has seen a decrease in burden over the past five years, apart from a limited outbreak in Colombo in March–April 2024. There were 95 cases in 2020 and a decrease year-over-year to 53 cases in 2023, reflecting an incidence rate of 0.24 per 100,000 population.

Overall improvement of quality of living standards has resulted in decreased typhoid incidence. More than 95 % of the population has access to safe drinking water and improved sanitary facilities. Typhoid vaccination, using the polysaccharide vaccine, is available for food handlers, who are considered a vulnerable group.

#### 1.4.13. Vietnam

Nguyen Thi Thu Huong presented updates on typhoid and TCV from Vietnam. Typhoid surveillance in Vietnam is conducted by the vaccine preventable diseases surveillance system within the EPI. Surveillance shows that the typhoid burden has decreased in recent years in Vietnam, with 242 cases in 2022, 112 cases in 2023, 70 cases in 2024, and 21 cases in the first three months of 2025. 80–90 % of cases have been observed in the South of Vietnam.

Vietnam introduced typhoid vaccine in 1997. [32] In 2024, typhoid was removed from the list of infectious diseases subject to the mandatory use of vaccines and medical biological products, and typhoid vaccine was removed from Vietnam’s EPI.

## 2. Discussion

Discussions during the 2024 Asia Regional Meeting on Typhoid and TCV focused on data-driven decision-making, diagnostic challenges, vaccine introduction drivers and barriers, political commitment, sustainable financing, and the role of WASH interventions. The following sections summarize the main takeaways and policy implications.

### 2.1. Data for decision-making

A variety of sources of typhoid burden data can be used to support vaccine introduction decisions. National decision-makers rely on burden of disease, vaccine cost-effectiveness, and EPI coverage data. Regional burden data is important for the prioritization of vaccines by RITAGs and WHO regional offices, especially as they balance prioritization of typhoid, malaria, HPV, and hexavalent vaccines. At the global level, Gavi uses burden data to forecast vaccine demand and allocate funding. Global data is also important for the WHO to inform their global recommendations and for funder organizations to prioritize research and implementation proposals. Each of these data sources has significant implications for health systems strengthening requirements, future financial implications of vaccine introduction decisions, and introduction strategies such as routine use and catch-up campaigns.

Multiple policymakers emphasized the need for more granular local burden data, disaggregated by sub-national regions, to inform introduction prioritization and strategies. Innovative tools are emerging to supply the burden data decision-makers need. Novel techniques, including serosurveys and genomics, demonstrate promise for integrated disease surveillance and can increase the geographic representativeness of data. Antibiotic resistance profiling and clinical surveillance for typhoid-associated intestinal perforation can also inform the value of vaccination.

### 2.2. Diagnostics for improved case detection

Country representatives called for improved diagnostics, both for point-of-care decision-making and appropriate antibiotic usage, and for surveillance purposes. While bone-marrow culture has the highest diagnostic accuracy, it is invasive and impractical in most settings and is not widely used in endemic regions. Blood culture, the current reference standard, is sub-optimal given its sensitivity of about 60 %, [33] requirement for highly-skilled technicians, decreased sensitivity following prior antibiotic exposure, and extensive infrastructure requirements, notwithstanding the costs of blood culture itself. Most sentinel surveillance occurs at tertiary care centers, which do not capture the full extent of disease burden. There have also been investments in molecular diagnostics, but these may not meet the desire for point-of-care tools that can influence diagnosis and treatment plans. Several lateral flow assays are in development and have been recently assessed, with mixed results. [34–36] With limited diagnostic tools, clinicians often rely on empirical antibiotic treatment, fueling inappropriate antibiotic use and the spread of AMR. Strengthening antimicrobial stewardship remains an important but challenging goal, and currently available diagnostics are unreliable for AMR surveillance. To that end, Gavi and the Combating Antibiotic Resistant Bacteria Biopharmaceutical Accelerator (CARB-X) announced funding schemes to support development of accurate point-of-care diagnostics for low-resource settings. [37]

### 2.3. Drivers and barriers of vaccine introduction

Attendees identified several factors that drive the impetus for TCV introduction:

- Rising awareness of typhoid as a major public health problem
- Stronger evidence on disease burden and potential impact of vaccination
- Data on economic burden of typhoid and cost-effectiveness of vaccination as a control strategy
- AMR as an urgent threat
- Gavi funding, available since 2017

Barriers to TCV introduction include competing vaccine priorities and gaps in data on the disease burden. Programmatic challenges also play a role, such as an increasingly crowded EPI schedule and the need for additional data on TCV co-administration with other routine vaccines such as Japanese Encephalitis.

Prior to vaccine introduction, sufficient lead time is required to analyze the implications of TCV introduction, including securing a NITAG recommendation; conducting financial, cold-chain logistics, and programmatic analysis; and securing the time, capacity, and resources to train health workers. [38] Countries are encouraged to explore opportunities to align TCV introduction with other immunization activities, such as co-administering TCV and other childhood vaccines or synchronizing a PCV-switch with TCV rollout.

### 2.4. Political will for vaccine introduction

Beyond local data, political will plays a critical role in TCV introduction. Countries that have successfully introduced typhoid vaccine or conducted campaigns have leveraged a combination of personal experience, economic concerns, outbreak responses, or AMR threats to drive decisions.

Participants discussed ways to strengthen communication between policymakers and health experts, improve surveillance data, and use cost-effectiveness studies to advocate for addition of TCV to national immunization schedules. Working groups explored potential funding strategies and technical support options. Cross-country collaboration and international partnerships were highlighted as key enablers to gain support for TCV introduction. While there is no universal approach to securing political buy-in, the international community and global health organizations can support local policymakers and vaccine champions with relevant data and advocacy tools.

Introducing TCV requires clear data, strong recommendations, and strategic communication. Nevertheless, TCV provides a lifesaving bridge to long-term solutions like clean water and sanitation infrastructure.

### 2.5. Sustainable immunization financing

Country representatives expressed concerns about balancing new vaccine introductions with sustaining high levels of coverage of existing vaccines. Several speakers stressed the need for sustainable financing models for immunization programs in the Asia region. For example, Lao PDR noted that budget cuts to their EPI have affected availability of vaccines already in the routine schedule.

For middle-income countries that have transitioned from Gavi support, sustainable financing of large immunization programs remains a major challenge. Participants proposed expanding Gavi’s remit to support vaccination programs in middle-income countries where large numbers of impoverished children reside. Others suggested leveraging innovative financing mechanisms for middle-income countries, such as collective bargaining with UNICEF, to secure vaccines at affordable costs.

Cost-effectiveness data were identified as a key factor influencing vaccine introduction decisions. However, delaying introduction of TCV in high-burden settings carries both health and economic costs. For example, Kenya’s introduction of TCV was approved and planned for 2024 but was delayed to 2025. Modeling suggests that this delay could result in 600,000 cases that would have been prevented by vaccine use. [39] TCV introduction in five African countries – Kenya, Burkina Faso, Ghana, Uganda, and Zambia – could reduce typhoid burden by 68–71 %, avert $46 million in treatment costs, and save thousands of lives in these countries. [40]

### 2.6. Clean water and sanitation engineering

Long-term typhoid control depends on investments in clean water and sanitation infrastructure. In rapidly urbanizing cities in Asia, intermittent water pressure during droughts or floods pulls surface contaminants into water systems, increasing infection risks. Urbanization trends may further drive typhoid transmission, as equitable access to sanitation services remains a challenge. In SEARO, approximately 85 % of the urban population has access to safely managed drinking water, and only 52 % has access to safely managed sanitation services [41].

Currently, Asia accounts for 54 % of the world’s urban population, with over 2.2 billion people. By 2050, this figure is projected to increase by 50 %. India alone is expected to add 416 million urban dwellers by 2050 [42]. Without increased access to safe water and investments in improved sanitation, we may see a backsliding on progress in typhoid incidence reductions.

Typhoid vaccines serve as a critical solution to protect populations from typhoid while broader water and sanitation improvements are implemented, and to provide protection when sanitation systems are disrupted by extreme weather events.

## 3. Conclusions

Impressive progress has been made in typhoid control in Asia. Novel surveillance tools are in development for enhanced disease and AMR tracking, while vaccine studies have demonstrated TCV’s efficacy, duration of protection, and the safety of co-administration with other routinely used vaccines. Policymakers are working across health and finance ministries to address vaccine financing and sustainability in countries transitioning from Gavi support.

Moving forward, typhoid control efforts must prioritize:

- Strengthening surveillance to better understand disease burden, vaccine impact, possible strain replacement, and AMR trends
- Developing reliable, low-infrastructure point-of-care diagnostic tests
- Improving antimicrobial stewardship practices to slow the spread of drug resistance
- Evaluating the durability of TCV protection and if there is a need for booster doses

Work should progress on all of these priorities. Failure to act will lead to preventable illness, rising costs to the health system, and further AMR escalation. Sustained efforts in vaccination, surveillance, and sanitation are critical to ensuring long-term typhoid control in Asia.

## Figures and Tables

**Table 1:** Progress on typhoid vaccine introduction in Asia and Oceania.

Typhoid Vaccine Introduction Progress	Countries
Introduced TCV	Nepal, Pakistan, Samoa
Advancing TCV Introduction	Bangladesh, India, Myanmar
Facing Financing & Competing Priority Barriers	Indonesia, Lao PDR, Philippines
Prioritizing Other Interventions	Bhutan, Sri Lanka, Malaysia, Vietnam
Successful TCV Campaigns	Fiji, Kiribati, Tuvalu

**Table 2:** Summary of participating country updates.

Country	Typhoid Incidence Rates^1^	Surveillance Trends	TCV Use	Vaccine Introduction Status and Challenges
Bangladesh	290 (234–370)	High incidence (913/100,000 in Dhaka); outbreak of ceftriaxone-resistant typhoid ongoing since May 2024.	2025 campaign	NITAG recommended TCV in 2023; introduction planned for 2025; inter-ministerial cooperation critical.
Bhutan	274 (210–350)	Low diagnosed typhoid case counts; decreasing stool culture positivity; 99.7 % safe drinking water coverage.	–	Vaccine introduction low priority; prioritize improving surveillance methods.
Fiji	51 (39–66)	Approx. 270 cases/year; incidence 31/100,000 in 2024; vaccine campaign faced hesitancy post-COVID.	2023 campaign	TCV introduced via campaign; challenges include outreach and scheduling.
India	262 (198–344)	High burden in cities; leading cause of bacteremia; emerging AMR; TCV effectiveness 83.7 % in Navi Mumbai.	Planning introduction	NITAG recommended TCV in 2022; Gavi support secured; surveillance and cost-effectiveness data prioritized.
Indonesia	169 (129–216)	Stable burden; public health interventions ongoing; competing priorities and budget constraints.	–	TCV medium priority; interest in local manufacturing; data needed for decisionmaking.
Lao PDR	167 (125–218)	Endemic in rural areas; burden decreased 2000–2017.	–	TCV low to moderate priority; budget and data gaps limit prioritization.
Malaysia	99 (75–131)	Decreasing incidence 2015–2024; high safe water/sanitation coverage.	–	Typhoid vaccines available privately; requests WHO guidance for low-incidence vaccination.
Myanmar	148 (112–191)	56 % cases in children under 15; typhoid a priority due to disaster risks.	Planning introduction	TCV high priority; included in 2025–2029 strategy; introduction planned for 2027.
Nepal	233 (181–302)	Variable incidence (400 to 1014 per 100,000); 7.7 million children vaccinated in 2022 campaign; 98 % routine coverage in 2023.	Introduced to routine immunization in 2022	Emphasis on hygiene promotion; TCV introduced for children 15 months of age.
Philippines	123 (94–160)	Increasing cases (15,873 in 2022 to 29,249 in 2024); limited blood culture availability; reliance on sanitation measures.	–	Developing NITAG and immunization strategy; cost and data reliability challenges.
Samoa	51 (38–66)	Mass campaign vaccinated ages 1–45; typhoid cases dropped from over 100 to fewer than 20 annually.	Introduced to routine immunization in 2021	TCV introduced with comprehensive typhoid elimination plan; focus on coverage and immunization strengthening.
Sri Lanka	97 (67–132)	Decreasing cases; incidence 0.24 per 100,000 in 2023; high access to safe water and sanitation.	–	Polysaccharide vaccine used for food handlers; overall burden reduced.
Vietnam	87 (67–114)	Decreasing cases; 242 in 2022 to 21 in early 2025; typhoid vaccine removed from EPI in 2024.	Removed from routine immunization in 2024	Surveillance ongoing; vaccine use discontinued in routine immunization.

Incidence rate estimates as of 2021 GBD reported in whole values per 100,000 person-years (95 % confidence intervals). Incidence rate data from IHME GBD [43].

## Data Availability

No data was used for the research described in the article.
